# Duodenectomy with jejunal advancement and reimplantation of the ampulla of Vater for recurrent right colon cancer: A case report

**DOI:** 10.1016/j.ijscr.2019.07.022

**Published:** 2019-07-19

**Authors:** Paul H. Sugarbaker

**Affiliations:** Program in Peritoneal Surface Malignancies, MedStar Washington Hospital Center, 106 Irving St., NW, Suite 3900, Washington, DC 20010, USA

**Keywords:** Local recurrence, Reoperative, Surgery, Pancreaticoduodenectomy, Whipple procedure

## Abstract

**Background:**

Local or regional recurrence of colon or rectal cancer frequently occurs if there is a positive margin of resection or spillage of cancer cells during the operation.

**Methods:**

The clinical course of a patient with right colon cancer recurrent within the resection site and on the anterior aspect of the duodenum was reviewed.

**Results:**

Resection of recurrent right colon cancer was accomplished by subtotal duodenectomy. Reconstruction was with jejunal advancement and reimplantation of the ampulla of Vater. No postoperative complications occurred and palliation was excellent.

**Conclusions:**

Prevention of recurrent right colon cancer is an import consideration for primary resection. However, if this duodenum is involved by recurrence, techniques for resection exist.

## Highlights

• A margin of resection for an advanced right colon cancer is the second portion of the duodenum.
• Recurrence of right colon cancer may involve the anterior aspect of the duodenum.
• Resection of the duodenum may occur without resection of the ampulla of Vater.
• Reimplantation of the ampulla of Vater into the proximal jejunum was accomplished without adverse events.

## 1 Introduction

Right colon cancer or appendiceal adenocarcinoma may progress immediately adjacent to the second and third portion of the duodenum. Because of direct invasion or tumor spillage at the time of right colon resection, disease recurrence on the adjacent structure may occur. Also, the progression of disease on the duodenum may occur in the absence of liver metastases, lymph nodal metastases, or extensive peritoneal metastases. In this clinical situation where systemic metastases have been excluded and all locally recurrent disease can be resected, a reoperative intervention may be considered. In order to optimally treat this clinical situation surgically, a procedure with clear margins and low incidence of adverse events should be selected. Resection in the absence of a pancreaticoduodenectomy (Whipple procedure) is preferred. In this case report I describe a procedure for an R-0 resection which may be an alternative to the Whipple procedure in selected patients. It requires only two anastomoses. It gives these patients an opportunity for prolonged survival with a short recovery time and excellent quality of life.

Data on this patient was prospectively recorded and then retrospectively reviewed at an academic institution. This research work has been reported in line with the SCARE criteria [1]. This study was registered as a case report on the www.researchregistry.com website with UIN 4798.

## 2 Patient presentation

August 2010, a 38 year old woman complained of two weeks of cramping abdominal pain. Colonoscopy showed an obstructing right colon cancer with circumferential involvement by the bowel wall. This was considered an en bloc resection by laparotomy. At the time of right colon resection the surgeon noted attachment of the right colon cancer to the duodenum. The tumor was dissected free with care and by pathology the margin was negative. Pathology showed a T4a cancer with a single positive lymph node; there was a total of 18 lymph nodes examined by the pathologist. The pathologist noted that the radial margin was positive. The tumor grade was III with poor differentiation and signet ring morphology. Twelve cycles of FOLFOX chemotherapy was administered without adverse events and without interruptions.

July 2011, a laparoscopy was performed and a biopsy positive for adenocarcinoma was obtained. CT showed a mass in the resection site and obstruction of the right ureter. A FOLFIRI regimen with Erbitux was initiated.

**Fig. 1:**
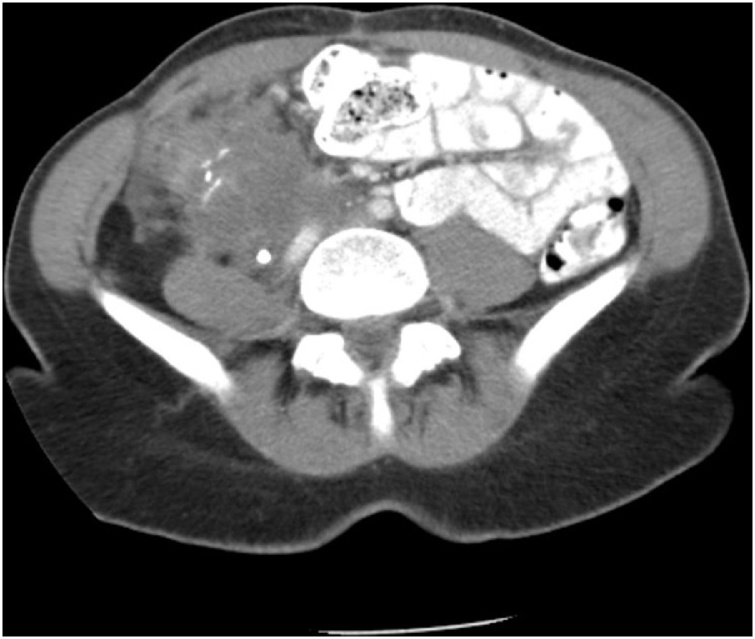
CT cut through the mid-abdomen in a 39 year old woman with recurrent right colon cancer. The staple line from the prior ileocolic anastomosis is in the central portion of the mass. A stent is in the obstructed right ureter.

September 2011, right-sided abdominal pain began increasing despite stenting of the right ureter. Physical examination showed a woman in moderate distress complaining of right-sided back and abdominal pain. No masses were palpable. A repeat CT showed a cancerous mass extending from right rectus muscle down to the psoas muscle. It was compressing the right ureter. The mass was immediately adjacent to the duodenum. No bowel obstruction was present (Fig. 1). No liver, lung, or retroperitoneal metastases were present. The patient was presented at the surgical oncology multidisciplinary team meeting and a plan for reoperation adopted. It was thought that the patient’s severe pain syndrome could be alleviated, at least in part, by a complete resection. The multidisciplinary team was aware that the possibility of a R1 resection was high.

**Fig. 2:**
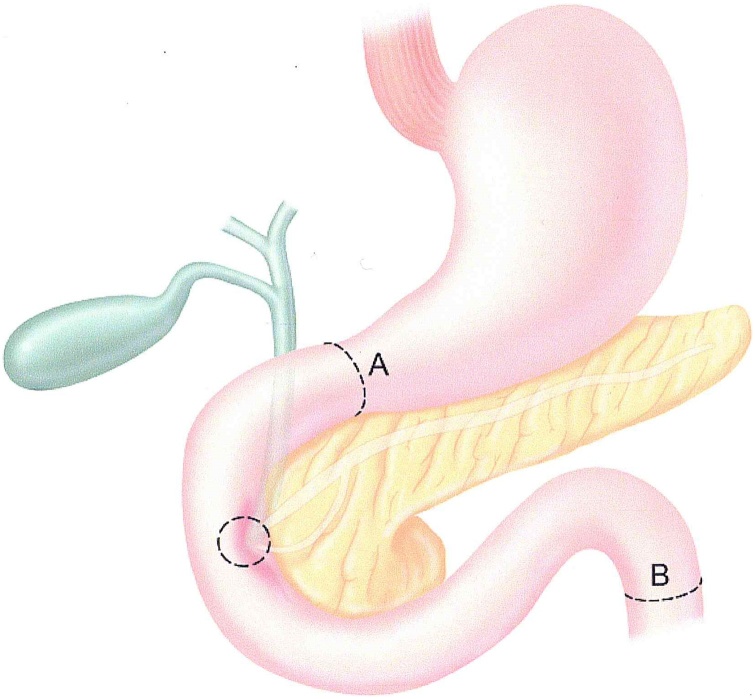
Total duodenectomy with detachment of the ampulla of Vater. Proximal transection at point A is distal stomach just proximal to the pylorus. Distal transection at point B is at the junction of fourth portion of the duodenum and jejunum.

September 8, 2011, exploratory laparotomy was performed which confirmed the absence of liver metastases and peritoneal metastases away from the right colon resection site. Resection required removal of the right kidney and ureter, peritonectomy of the right paracolic sulcus, redo right colon resection with greater omentectomy. Cancer was layered out over the right lateral and anterior aspect of the second and third portion of the duodenum. Complete removal of the duodenum was performed with sparing of a 3 cm patch of duodenal wall around the ampulla of Vater. The duodenum was opened widely anteriorly to separate the spared ampulla from the remainder of the duodenum. All cancer was resected with a positive margin (R-1 resection) on the right common iliac artery. The resection required 6 units of packed red blood cells and 4 units of fresh frozen plasma. The limits of the duodenal resection are shown in Fig. 2.

**Fig. 3:**
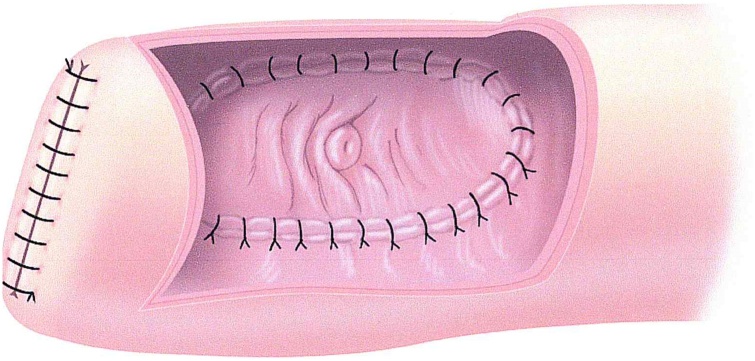
Ampulla of Vater is jejunal anastomosis. After the enterotomy in the jejunum is made, a line of sutures inferiorly is made with absorbable sutures tied on the inside of jejunum. Superiorly, the sutures are placed to be tied outside the jejunum.

**Fig. 4:**
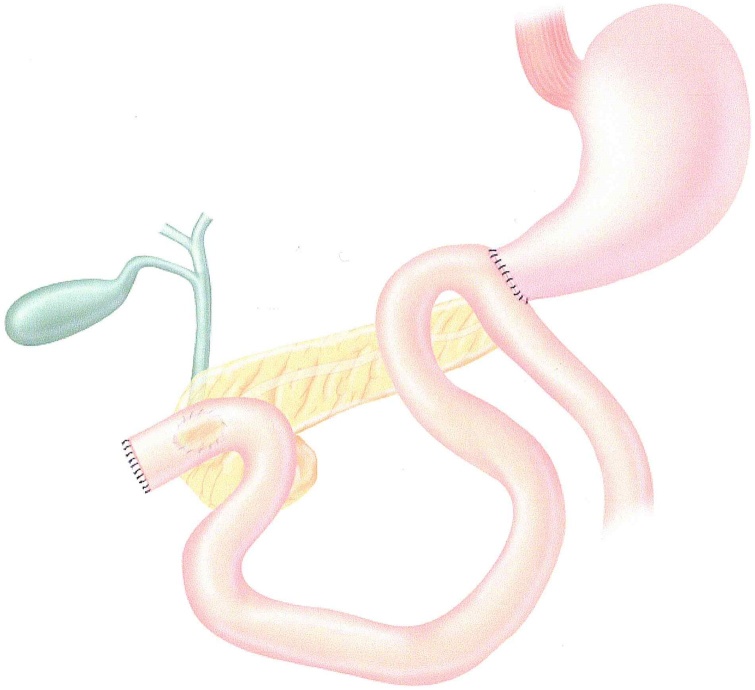
An anastomosis of end of stomach to antimesenteric border of jejunum completes the reconstruction.

To reconstruct a routine ileocolic anastomosis was performed with end of proximal colon sutured to antimesenteric aspect of the terminal ileum. The proximal jejunum was advanced and its end turned in with sutures over the staple line. A jejunostomy the width of the patch of ampulla of Vater was made. The inferior aspect of the periampullary duodenal edge was sutured to the jejunum with individual 3-0 Maxon suture material (Covidien, Minneapolis, MN). The knots from this line of sutures was on the inside of the jejunum (Fig. 3). The superior line of sutures joining the periampullary duodenum to jejunum was interrupted sutures with the knots on the peritoneal aspect of the jejunum. To complete the reconstruction the antimesenteric aspect of the jejunum was sutured to the stomach just proximal to the pylorus (Fig. 4). The surgery required 10 h.

Postoperatively, the patient’s pain syndrome remained difficult to manage. An upper extremity deep vein thrombosis was treated by Coumadin (warfarin sodium) anticoagulation. The patient remained in the hospital on total parenteral nutrition for 23 days. The patient died of progressive disease after 16 months. Disease recurrence was limited to the pelvis causing bowel obstruction. No liver metastases occurred.

## 3 Discussion

The first issue that should be considered in this patient is the management of the obstructing right colon cancer. Colonoscopy showed a circumferential mass. Also, the primary tumor was abutting the duodenum. The pathology report showed cancer present at the margin of resection and depth of invasion was T4. These are all poor prognostic signs [2]. Although a cause of the patient’s extensive local recurrence is impossible to determine with certainty, a likely event was clearance of the primary cancer but a failure of its containment. Cancer cells may have been widely distributed, as a result of surgical trauma, to all the margins of resection of a right colon resection. The abdominal-pelvic surfaces at risk would include the peritoneum of the right paracolic sulcus, the right ureter and right common iliac artery, and the anterior surface of the duodenum. These are the anatomic sites from which recurrent cancer was resected even though these anatomic sites were thought to be free of cancer at the completion of the right colon resection. There are treatment strategies that promise some success in the management of minimal residual disease [3,4]. Sugarbaker and coworkers were convinced that the optimal time to prevent intraoperative tumor dissemination was at the time of primary cancer resection. Delay would allow viable cancer cells to be covered by the wound healing process [5]. Another strategy to optimally manage a poor prognosis colon cancer is systematic second-look [6,7]. Reliance on systemic chemotherapy to control resection sites contaminated by cancer cells will always be disappointing.

Unfortunately, right colon cancer often presents in an advanced stage so that complete containment with removal of the malignant process does not occur. This tumor cell contamination of the operative site may be from cells dislodged from the primary cancer itself. This is a prominent occurrence with T3 or T4 cancers, as in the patient presented here. Also, transected lymphatic channels can leak lymph contaminated by cancer cells into the resection site. Similarly, cancers that show venous invasion may contaminate the resection site if there is blood loss into the abdomen or pelvis [5]. Any or all of these causes of cancer cell dissemination within the resection site may occur. Seeding with high density is common within the resection site while low density seeding on distant serosal surfaces may eventuate as peritoneal metastases [6,7].

The surgical strategy of duodenal resection with detachment of the ampulla of Vater and a surrounding patch of duodenum has been previously described for benign disease. Kavlie et al. reported a successful total duodenectomy, detachment of the ampulla of Vater, and its reimplantation into the proximal jejunum [8]. This was for an extensive bleeding hemangioma of the duodenum. They anastomosed stomach to end of proximal jejunum and then ampulla to side of jejunum. In our patient the end of jejunum was closed, the ampulla anastomosed to side of jejunum and then a gastrojejunostomy performed. In my opinion, exclusion of the ampulla to jejunum anastomosis from enteric contents in the postoperative period is a superior reconstruction.

Williamson et al. reported resection of the first and second portions of the duodenum along with a portion of the pancreatic head. The ampulla was anastomosed to a Roux loop [9]. Fleisch reported resection of the first and second portions of the duodenum with detachment of the ampulla. Reconstruction was with the interposition of a jejunal segment to anatomically reconstruct the duodenum. The jejunal segment and ampulla were anastomosed [10]. Young et al. reported duodenectomy with reimplantation of the ampulla of Vater into a Roux loop for the successful treatment of megaduodenum [11].

In the case report recorded in this manuscript, the first and second portions of the duodenum were extensively invaded by cancer and required resection. I proceeded to remove the normal third and fourth portions of the duodenum in order to simplify the reconstruction. The advancement of the closed proximal jejunum to the patch of ampulla of Vater allowed an anastomosis with no tension. A gastrojejunostomy a full 40 cm distal completed the reconstruction. This strategy for resection of duodenal invasion by primary or right colon cancer is, I think, an alternative to a pancreaticoduodenectomy. A pancreaticojejunal anastomosis as required in a Whipple procedure is avoided and replaced by an ampulla to jejunal anastomosis [12]. Also, the choledochal-jejunal anastomosis is avoided. The simplicity of the total duodenectomy, jejunal advancement and reimplantation of the ampulla makes is a surgical strategy that may be considered as an alternative to the Whipple procedure in selected patients.

## Sources of funding

Data management and secretarial support provided by Foundation for Applied Research in Gastrointestinal Oncology.

## Ethical approval

Local IRB-approval for this case report was not required:

MedStar Health Institutional Review Board has determined that a case report of less than three (3) patients **does not meet the DHHS definition of research** (45 CFR 46.102(d)(pre-2018)/45 CFR 46.102(l)(1/19/2017)) **or the FDA definition of clinical investigation** (21 CFR 46.102(c)) and therefore are not subject to IRB review requirements and **do not require IRB approval**.

This case report is of 1 patient.

## Consent

Written and signed consent was obtained from the patient.

## Author contribution

Paul H. Sugarbaker, MD: study concept or design, data collection, data analysis or interpretation, writing the paper.

## Registration of research studies

This study was registered as a case report on the www.researchregistry.com website with UIN 4798.

## Guarantor

Paul H. Sugarbaker, MD.

## Provenance and peer review

Not commissioned, externally peer-reviewed.

## Declaration of Competing Interest

Paul H. Sugarbaker has no conflicts of interest to declare.
